# Correction: Association of zinc deficiency with infectious complications in pediatric hematopoietic stem cell transplantation patients

**DOI:** 10.1371/journal.pone.0359637

**Published:** 2026-09-29

**Authors:** Warangkhana Suwanphoerung, Chompunuch Klinmalai, Sasivimol Rattanasiri, Samart Pakakasama, Usanarat Anurathapan, Suradej Hongeng, Nalinee Chongviriyaphan, Nopporn Apiwattanakul

In the Patients and Methods subsection of the Abstract, there is an error in the first sentence. The correct sentence is: Thirty patients undergoing HSCT at Ramathibodi Hospital during May 2020–September 2021 were enrolled.

In the Study design and patients subsection of the Methods, there is an error in the first sentence of the first paragraph. The correct sentence is: Pediatric patients aged 0–18 years old who underwent HSCT at Ramathibodi Hospital in Thailand during May 2020–September 2021 were enrolled in this study.
