# Supplementary material for: Association of zinc deficiency with infectious complications in pediatric hematopoietic stem cell transplantation patients
Source: PLoS One. 2022 Dec 27;17(12):e0279439. doi: 10.1371/journal.pone.0279439 (PMC9794056; doi:10.1371/journal.pone.0279439)
Supplement: S2 File — (DOCX) [file pone.0279439.s002.docx]

**Document for donors of stem cells**

Date ………………………………………………………………………… Number of donation ………………………………………..

Name ………………………………………………………………………. Identification number ………………………………………

Sex 🞏 male 🞏 female Age …………………………… years Date of birth …………………………………………………….

Body weight ………………………………. Kg Height ……………………………………… cm Blood group …………………..

**Exclusion criteria for donation**

**General information Yes No General information Yes No**

1. Sexual risks 15. Bleeding tendency 🞏 🞏

1.1. Polygamy 🞏 🞏 16. Chronic cough, hemoptysis, 🞏 🞏

1.2. Homosexuality 🞏 🞏 asthma

2. History of sexual transmitted 🞏 🞏 17. Allergic rhinitis 🞏 🞏

Disease (STD) 18. Epilepsy 🞏 🞏

3. Got out of jail within 3 years 🞏 🞏 19. Chronic dermatitis, DM, TB 🞏 🞏

4. Intravenous drug abuse 🞏 🞏 Heart/kidney/thyroid diseases, HT 🞏 🞏

5. Intoxicated 🞏 🞏 20. Tumor or cancer 🞏 🞏

6. Unintentional weight loss 🞏 🞏 21. Frequent syncope 🞏 🞏

In 3 months 22. Currently having common cold 🞏 🞏

7. Went to malaria endemic area 🞏 🞏 23. History of arrhthymia 🞏 🞏

within 1 year or had malaria  **History of medications and vaccinations**

within 3 years 24. Aspirin/muscle relaxants/NSAIDS 🞏 🞏

8. Diarrhea within 7 days 🞏 🞏 25. Vaccinations or receiving serum 🞏 🞏

9. Dental procedures 🞏 🞏 within 14 days

within 3 days 26. Participation in HIV vaccine project 🞏 🞏

10. Major surgery within 🞏 🞏 Only for female

6 months or minor surgery 27. Currently having menstruation 🞏 🞏

within 7 days …………………. 28. Currently pregnant 🞏 🞏

11. Received blood transfusion 🞏 🞏 29. Currently lactating 🞏 🞏

within 1 year 30. Child delivery or abortion for 🞏 🞏

12. Lived in England during 🞏 🞏 past 3 months

1980-1996 for at least 6 months

13. History of tattooing 🞏 🞏

14. Recovered from hepatitis or 🞏 🞏

family of hepatitis

**Donating to patient Name ……………………………………………….. Hospital number ………………………………….**

**Consent for stem cell donation**

I am hereby voluntarily donating my stem cells to the aforementioned patient. For this donation process, I have read and understood the information regarding the exclusion from donation. I certify that all above information is true.

**Signature of donor** …………………………………..
